# Editorial: Proceedings From the 4th Memorial Alicia Pueyo Workshop: “Moving Towards a Cure for Diffuse Intrinsic Pontine Glioma”

**DOI:** 10.3389/fonc.2019.00042

**Published:** 2019-01-31

**Authors:** Jaume Mora

**Affiliations:** Hospital Sant Joan de Déu Barcelona, Barcelona, Spain

**Keywords:** DIPG, diffuse intrinsic pontine glioma, CED = convection-enhanced delivery, BBB drug transport, cell in origin, cns immunotherapy

Diffuse Intrinsic Pontine Glioma, DIPG, is yet a devastating and incurable children's brain tumor. In 2006 Alicia was diagnosed of DIPG at age 6 years. Impossible to remove by surgery, and with no effective treatments except for palliative radiotherapy to gain a few more months, then like today, there is no cure, and life expectancy remains about 12–18 months. Alicia didn't break the odds and after 15 months of fight, she succumbed to the disease in November of 2007. From that moment, the family like many others who have gone through this nightmare strongly committed to fight this terrible disease to find a cure. The Alicia Pueyo Fund was set-up in 2008 at Sant Joan de Déu Hospital, Barcelona. The aims of the fund were providing support and information to affected families and facilitate international collaboration between researchers in the field. The challenge was up to those willing to change minds, to think out of the box and to walk new paths to cure DIPG. The first ever International Workshop in DIPG, the first Memorial Alicia Pueyo meeting, took place in our place, Barcelona, in February 26, 2009. After the first meeting, the international network of families prompted subsequent meetings in Toronto, Cincinnati, and Amsterdam. The second Memorial Alicia Pueyo took place February 26, 2012. The DIPG collaboration networks got stronger both in the US and Europe and International registries were set up. The Alicia Pueyo's Fund focused on supporting the Hospital Sant Joan de Déu research group in DIPG. In 2012, with the invaluable collaboration of the Necker Institute in Paris, the first patient was sent from Barcelona to Paris to undergo biopsy. Since April 2013, biopsy is a standard procedure for DIPG patients at Hospital Sant Joan de Déu. The availability of tissue samples for research brought a completely different scenario. Researchers were able to identify DIPG mutations and understand why treatments failed. DIPG was defined as a biological entity by itself, different to any other adult or pediatric brain tumor. The 2016 WHO classification of brain tumors has recognized the entity as midline gliomas with H3K27M. Robust mouse models of DIPG and several cell lines were rapidly developed, tools that have accelerated enormously the development of novel drugs and delivery methods. The fantastic acceleration of DIPG knowledge was discussed at the third Memorial Alicia Pueyo Workshop that took place in February 26, 2015. New clinical trials incorporating biological biomarkers were presented. Since then the International networks of researchers have reported several outstanding papers understanding the cellular origin of DIPG, the epigenome derived from the Histone H3 founding mutation and the vascular and immune microenvironment characteristic of DIPG.

Current knowledge, international alliance, laboratory models, and dedicated clinical trials were unthinkable when Alicia was diagnosed 12 years ago. The extraordinary commitment of affected families and the coordinated work of clinicians and researchers willing to change minds have prompted revolutionary advances to find a cure for DIPG. The IVth Alicia Pueyo International Workshop took place March 12–13, 2018. Some of the relevant presentations of the meeting are summarized in this special Research Topic collection. The Meeting was so successful that paper summaries collected in this series have attracted more than 7,000 reads in less than a year.

The first part of the meeting covered several fundamental biology aspects regarding cell of origin, structure and microenvironment including presentations from NADA JABADO (McGill University, Montreal, Quebec, Canada) on Spatiotemporal Homogeneity; MICHELLE MONJE (Department of Neurology, Stanford University.) on fundamental neurobiology that applies to DIPG development; ESTHER HULLEMAN (VU University Medical Center, Amsterdam) on EMT programs; and culminated with MARIELLA FILBIN (Dana-Farber Cancer Institute. Boston) who presented for first time the single cell sequencing analysis of DIPG biopsies revealing oligodendrocyte precursor cells as the cell of origin for DIPG. The second part described the current DIPG management and ongoing clinical Trials. Presentations included those from the HSJD group with SONIA PACO describing the immune microenvironment from patients treated in the Phase I clinical trial that ANDRES MORALES (Pediatric Neuro-Oncology. HSJD Barcelona) described using dendritic cell vaccination for newly diagnosed DIPG, paper included in this Frontiers collection. LORENZA GANDOLA (Istituto Nazionale dei Tumori, Milan) presented the promising radiotherapy approach developed in Milan; DANNIS VAN VUURDEN (VU University Medical Center, Amsterdam) summarized the many different SIOP-BTG strategies; MARK KIERAN (Dana-Farber Cancer Institute, Boston) presented the final results from the BATS DIPG study, a multiinstitutional clinical trial completed in the USA for first time requiring biopsy of the tumor; JACQUES GRILL (Institut de cancérologie Gustave Roussy, Paris) summarized the many vicissitudes and evolutions of the ongoing BIOMEDE study in France and other European countries. Finally, MARK M. SOUWEDAINE (Weill Medical College of Cornell University, NY) presented data from the Phase I trial from MSKCC/Cornell using Convection-enhanced delivery (CED) of antibody 8H9. The last section of the meeting explored the many novel targets investigated in laboratories worldwide. Presentations included CHRIS JONES (The Institute of Cancer Research, Sutton, UK) who described the many opportunities targeting ACVR1; KATHY WARREN (Pediatric Oncology Branch, NCI. USA) who explored the difficulties that the blood-brain barrier (BBB) poses to the pharmacokinetics of DIPG, a paper summary available in this Frontiers collection. As a potential novel approach to surmount the BBB, Meritxell Teixidó (Institut de Recerca Biomèdica Barcelona) presented the basic chemistry of Nanocarriers for BBB shuttling. OREN BECHER (Northwestern University, Chicago) reported on the CDK4/6 and PDGF-B pathways, and its potential for future treatments, paper available in this series. MICHELLE MONJE disclosed the controversial and pre-clinically impressive results using anti-GD2 therapy for DIPG; ESTHER HULLEMAN showed the opportunities of Preclinical platforms to evaluate CED and MARTA ALONSO (CIMA. Universidad de Navarra. Pamplona. Spain) briefly summarized the ongoing Phase I trial using Oncolytic virotherapy. Finally, ANGEL M. CARCABOSO (Developmental tumor Laboratory. HSJD) discussed the different drugs being tested in the laboratory able to penetrate the CNS and with migration inhibitory features that can change the natural history of DIPG preclinical models.

In less than 10 years since the first Alicia Pueyo meeting, the progress in the field of DIPG has been so impressive, that one can envision the first signs of cure approaching in the horizon of science. This collection is a tribute to all the Alicia's and families worldwide that have committed their efforts and energies to solve this medical challenge.

## Author Contributions

The author confirms being the sole contributor of this work and has approved it for publication.

### Conflict of Interest Statement

The author declares that the research was conducted in the absence of any commercial or financial relationships that could be construed as a potential conflict of interest.

